# Cardiac preconditioning with sphingosine-1-phosphate requires activation of signal transducer and activator of transcription-3

**DOI:** 10.5830/CVJA-2014-016

**Published:** 2014-06

**Authors:** Roisin F Kelly-Laubscher, Jonathan C King, Damian Hacking, Sarin Somers, Samantha Hastie, Tessa Stewart, Aqeela Imamdin, Gerald Maarman, Sarah Pedretti, Sandrine Lecour

**Affiliations:** Hatter Institute for Cardiovascular Research in Africa, Chris Barnard Building, Medical School Campus, University of Cape Town, Cape Town, South Africa; Hatter Institute for Cardiovascular Research in Africa, Chris Barnard Building, Medical School Campus, University of Cape Town, Cape Town, South Africa; Hatter Institute for Cardiovascular Research in Africa, Chris Barnard Building, Medical School Campus, University of Cape Town, Cape Town, South Africa; Hatter Institute for Cardiovascular Research in Africa, Chris Barnard Building, Medical School Campus, University of Cape Town, Cape Town, South Africa; Hatter Institute for Cardiovascular Research in Africa, Chris Barnard Building, Medical School Campus, University of Cape Town, Cape Town, South Africa; Hatter Institute for Cardiovascular Research in Africa, Chris Barnard Building, Medical School Campus, University of Cape Town, Cape Town, South Africa; Hatter Institute for Cardiovascular Research in Africa, Chris Barnard Building, Medical School Campus, University of Cape Town, Cape Town, South Africa; Hatter Institute for Cardiovascular Research in Africa, Chris Barnard Building, Medical School Campus, University of Cape Town, Cape Town, South Africa; Hatter Institute for Cardiovascular Research in Africa, Chris Barnard Building, Medical School Campus, University of Cape Town, Cape Town, South Africa; Hatter Institute for Cardiovascular Research in Africa, Chris Barnard Building, Medical School Campus, University of Cape Town, Cape Town, South Africa

**Keywords:** STAT-3, cardioprotection, preconditioning, sphingosine-1-phosphate, myocardial infarction

## Abstract

**Aims:**

Sphingosine-1-phosphate (S1P) is a cardioprotective agent. Signal transducer and activator of transcription 3 (STAT-3) is a key mediator of many cardioprotective agents. We aimed to explore whether STAT-3 is a key mediator in S1P-induced preconditioning.

**Methods:**

Langendorff-perfused hearts from Wistar rats and wild-type or cardiomyocyte-specific STAT-3 knockout mice were pre-treated with S1P (10 nmol/l), with or without the STAT-3 pathway inhibitor AG490, before an ischaemia–reperfusion insult. Triphenyltetrazolium chloride and Evans blue staining were used for the determination of infarct size. Western blot analysis was carried out on the S1P pre-treated hearts for detection of cytosolic, nuclear and mitochondrial phosphorylated and total STAT-3 proteins.

**Results:**

Pre-treatment with S1P decreased the infarct size in isolated rat (5 ± 3% vs control 26 ± 8%, *p* < 0.01) and wild-type mouse hearts (13 ± 1% vs control 33 ± 3%, *p* < 0.05). This protective effect was abolished in the rat hearts pre-treated with AG490 (30 ± 10%, *p* = ns vs control) and in the hearts from STAT-3 knockout mice (35 ± 4% vs control 30 ± 3%, *p* = ns). Levels of phosphorylated STAT-3 were significantly increased in both the nuclear (*p* < 0.05 vs control) and mitochondrial (*p* < 0.05 vs control) fractions in the S1P pre-treated hearts, but remained unchanged in the cytosolic fraction (*p* = ns vs control).

**Conclusion:**

These novel results demonstrate that pharmacological preconditioning with S1P in the isolated heart is mediated by activation of mitochondrial and nuclear STAT-3, therefore suggesting that S1P may be a novel therapeutic target to modulate mitochondrial and nuclear function in cardiovascular disease in order to protect the heart against ischaemia–reperfusion.

## Abstract

Signal transducer and activator of transcription 3 (STAT-3) is a downstream mediator of many cardioprotective agents, most notably, of ischaemic pre- and postconditioning,^1^-^5^ i.e. protection brought about by repeated bouts of brief ischaemia before (preconditioning) and after (postconditioning) a prolonged period of ischaemia. Many pharmacological conditioning agents such as adenosine,^6^ opioids,^7^ erythropoietin,^8^ ethanolamine,^9^ melatonin,^10^ leptin,^11^ and tumour necrosis factor alpha (TNFα)^4^,^12^ also protect via the activation of STAT-3.

These findings have led to the description of a novel pathway involved in both mechanical and pharmacological preconditioning: ‘survivor activating factor enhancement’ (SAFE).^5^,^13^ This study focused on the role of the SAFE pathway, more specifically STAT-3, in S1P-induced preconditioning.

Sphingolipids and their metabolites are important signalling molecules in the heart. There is growing evidence that major components of the sphingolipid pathway, such as ceramide, sphingosine and sphingosine-1-phosphate (S1P) can protect the heart against an ischaemia–reperfusion insult, but the exact mechanism remains unclear.^14^-^18^ Signalling molecules such as protein kinase Cε,^15^ the pro-survival protein kinase-B/Akt^18^ and extracellular signal-regulated kinase 1/2, which are major components of the ‘reperfusion injury salvage kinase’ pathway (RISK),^19^,^20^ are implicated in S1P-induced preconditioning.

Recent data have demonstrated that S1P upregulates STAT-3 phosphorylation in other organ systems both in vitro^21^ and *in vivo*.^22^ It was also demonstrated that STAT-3 mediates S1P-induced protection against doxorubicin-induced toxicity in isolated ventricular cardiomyocytes.^23^ Similarly, pharmacological postconditioning with S1P protects isolated mouse hearts against a global ischaemia–reperfusion insult via STAT-3 activation in the mitochondrion and nucleus,^24^ therefore suggesting a link between S1P and STAT-3, and hence activation of the SAFE pathway by S1P. However whether the same mechanism of protection is involved in S1P-induced preconditioning remains unknown.

In this study, we used cardiomyocyte-specific STAT-3 knockout mice and a STAT-3 pathway inhibitor to investigate the role of STAT-3 in the cardioprotective effect of pharmacological preconditioning with S1P against both global and regional ischaemia–reperfusion injury.

## Methods

All experimental procedures were performed with the approval of the Faculty of Health Sciences Animal Ethics Committee, University of Cape Town. All protocols were carried out in compliance with the European Convention for the Protection of Vertebrate Animals used for Experimental and other Scientific Purposes (Council of Europe No 123, Strasbourg 1985).

Male Wistar rats (250–300 g, *n* = 56), wild-type and cardiomyocyte-specific STAT-3 knockout mice (14–16 weeks, *n* = 31) were bred and obtained from the University of Cape Town Animal Unit as previously described.^6^

## Isolated STAT-3 knockout heart model

Cardiomyocyte-specific STAT-3 knockout mice (STAT-3 KO) and wild-type littermate control mice were anaesthetised (sodium pentobarbitone, 60 mg/kg i.p.) and heparinised (25 IU i.p.). Once an adequate level of anaesthesia was achieved, the chest was opened, the heart was rapidly removed and placed in ice cold (4oC) modified Krebs-Henseleit buffer, and the aorta was cannulated.

The hearts were then perfused with Krebs-Henseleit buffer using the Langendorff system as previously described.^25^ A minimum of 1.5 ml/min and maximum of 5.0 ml/min of coronary flow rate, heart rate between 460 and 600 beats per minute (bpm) and developed force ≥ 4 g was deemed acceptable. No haemodynamic data were collected during the protocol.

After a 20-minute stabilisation period, the hearts were subjected to 35 minutes of global ischaemia followed by 45 minutes of reperfusion. Hearts were pre-treated with S1P (10 nmol/l in DMSO) for seven minutes, followed by a 10-minute washout period before global ischaemia, as previously described.^14^ At the end of each experimental protocol, the infarct size was assessed by triphenyltetrazolium chloride (TTC) staining. The infarct size was determined with planimetry.^25^

## Isolated rat heart model

The rats were anaesthetised with sodium pentobarbital (50 mg/kg i.p.) and heparinised (500 IU i.v.). The hearts were rapidly excised and perfused retrogradely by the Langendorff technique, as previously described.^25^ Rat hearts that did not comply with the following criteria were excluded: (1) left ventricular pressure greater than 80 mmHg, (2) coronary flow rate at a minimum of 8 ml/min and maximum of 16 ml/min, (3) heart rate at a minimum of 240 bpm and maximum of 400 bpm.

**Fig. 1.**
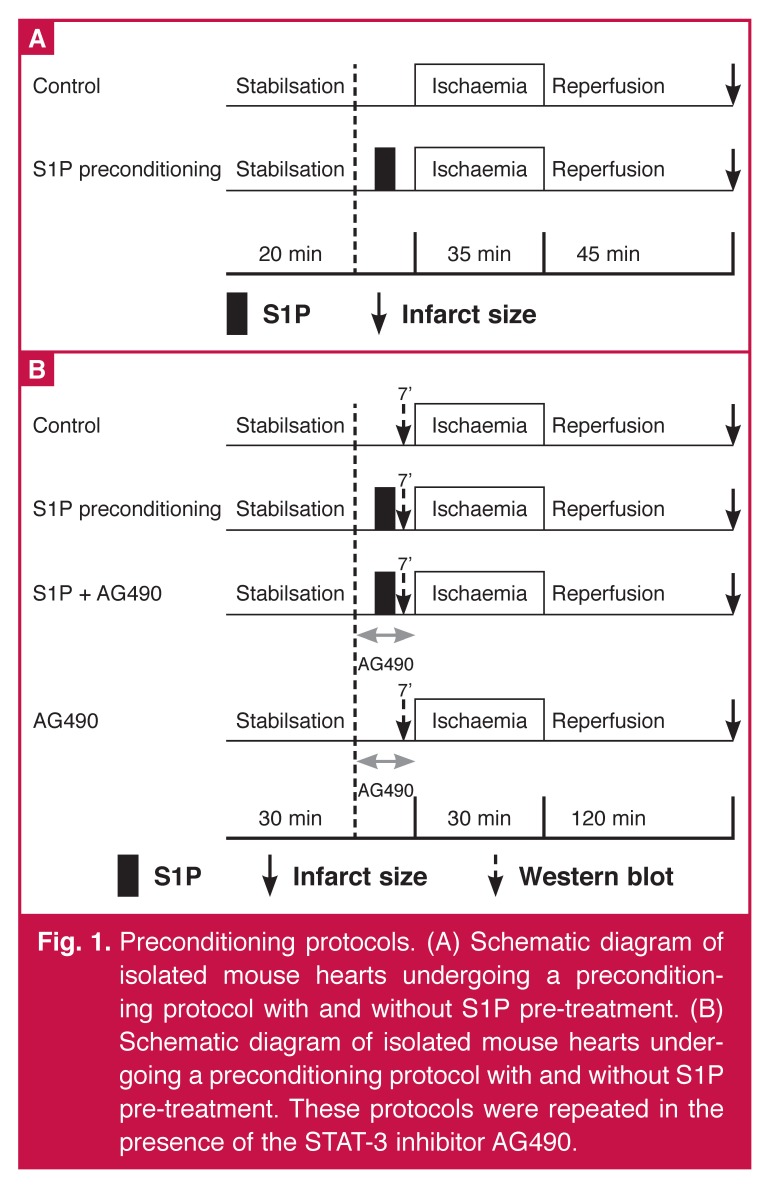
Preconditioning protocols. (A) Schematic diagram of isolated mouse hearts undergoing a preconditioning protocol with and without S1P pre-treatment. (B) Schematic diagram of isolated mouse hearts undergoing a preconditioning protocol with and without S1P pre-treatment. These protocols were repeated in the presence of the STAT-3 inhibitor AG490.

After 30 minutes of stabilisation, all hearts were subjected to 30 minutes of regional standard ischaemia by occlusion of the left coronary artery and 120 minutes of reperfusion, as previously described.^25^ Hearts were pre-treated with S1P (10 nmol/l in DMSO) for seven minutes followed by a 10-minute washout period before the standard ischaemia. In half of the rats, the JAK-STAT-3 inhibitor, AG490 (100 nmol/l),^26^ was given for 15 minutes: three minutes before, seven minutes concomitantly with S1P (S1P + AG490 group) and five minutes after perfusion with S1P (Fig. 1).

Haemodynamic parameters were assessed throughout the experiment and included heart rate, left ventricular developed pressure (LVDP) and coronary flow. Haemodynamic variables were statistically tested for intergroup and intragroup variation. For the measurement of infarct size, the coronary artery was re-occluded at the end of the reperfusion period and a solution of 2.5% Evans blue was perfused to delineate the area at risk (AAR).

The hearts were then frozen and cut into slices, and incubated in sodium phosphate buffer containing 1% w/v TTC for 15 minutes to visualise the unstained infarct region. The infarct size and AAR were determined with planimetry and the infarct size was expressed as a percentage of the AAR.

## Preparation of hearts for Western blots

In the isolated rat hearts, the ventricular tissue from control and S1P pre-treated hearts was excised before the regional ischaemic insult (seven minutes after S1P treatment), freeze clamped using Wollenberger tongs in liquid nitrogen and stored at –80°C. The frozen hearts were wrapped in aluminium foil and pulverised under liquid nitrogen before being transferred to tubes for storage.

For extraction of nuclear and cytosolic protein, pieces of the left ventricle were homogenised twice by Polytron using the homogenisation buffer described by Williams and Ford.^27^ The suspension was then centrifuged at 10 000 *g* (12 000 rpm) for five minutes at 4°C. The supernatant containing the cytosolic fraction was collected and transferred into a fresh tube. The pelleted fraction was resuspended in the same homogenisation buffer supplemented with 1% Triton X-100, as described previously.^27^ After centrifugation for 30 minutes at 10 000 g (12 000 rpm) at 4°C, the supernatant containing the nuclear fraction was carefully removed and transferred to a clean tube.

For extraction of mitochondrial and cytosolic protein, the frozen rat hearts were finely minced with scissors in a lysis buffer, as described by Lewin *et al.*,^28^ and then transferred to a Dounce homogeniser. After homogenisation, the suspension was centrifuged at 600 *g* for five minutes at 4°C. The supernatant was transferred to a fresh micro-centrifuge tube and centrifuged at 10 300 *g* (11 500 rpm) for 10 minutes. The supernatant is now the cytosolic fraction and the pellet the mitochondrial fraction.

The pellet was resuspended in 40 µl incubation buffer (250 mM sucrose, 25 mM Tris, 8.5 mM KH_2_PO_4_). The proteins were quantitated and an equal volume low-ionic strength sample buffer [10 % sodium dodecyl sulphate (SDS), glycerol, mercaptoethanol, Tris (pH 6.8), bromophenol blue) was added to each sample.

## Western blot analysis

Phosphorylated and total STAT-3 levels were analysed by SDS polyacrylamide gel electrophoresis with antibodies from Cell Signalling Technology. Proteins were revealed with enhanced chemiluminescence (ECL) Western blotting detection reagents (Amersham, UK) and the images were captured electronically using a GeneGnome HR (Syngene Bioimaging, UK).

Levels of phosphorylated and total STAT-3 were determined in the same samples and under the same conditions but on separate membranes. Equal loading was verified with β-actin staining for the nuclear and cytoplasmic fractions and voltage-dependent anion channel (VDAC) for the mitochondrial fractions. Levels of phosphorylated proteins were normalised to their total protein levels.

Relative densitometry was determined using Quantity One software (Biorad). The cytoplasmic fraction analysed in these experiments came from a different group of hearts, however all hearts came from the same strain of rat of the same age and they were treated identically.

## Statistical analysis

Data are presented as mean ± SEM. Comparisons between multiple groups were performed by one-way ANOVA followed by the Dunnet’s post hoc test (Graph Pad Instat). A value of *p* < 0.05 was considered statistically significant.

## Results

## S1P-induced preconditioning was inhibited in the STAT-3 knockout mice

**Fig. 2.**
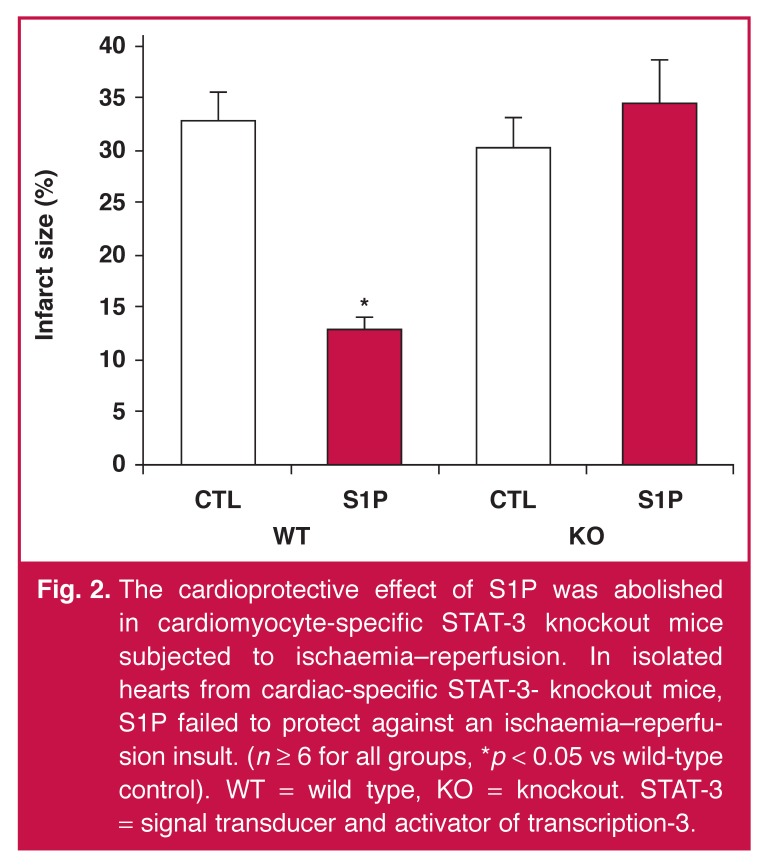
The cardioprotective effect of S1P was abolished in cardiomyocyte-specific STAT-3 knockout mice subjected to ischaemia–reperfusion. In isolated hearts from cardiac-specific STAT-3- knockout mice, S1P failed to protect against an ischaemia–reperfusion insult. (*n* ≥ 6 for all groups, *p < 0.05 vs wild-type control). WT = wild type, KO = knockout. STAT-3 = signal transducer and activator of transcription-3.

Control hearts subjected to 35 minutes of global ischaemia and 45 minutes of reperfusion had an infarct size of 33 ± 3%. Pre-treatment with S1P (10 nmol/l) resulted in a significant reduction in the infarct size to 13 ± 1% (Fig. 2) (*p* < 0.05 vs wild-type control hearts). Ischaemic control hearts from STAT-3 knockout mice had an infarct size of 30 ± 3 %. The infarctsparing effect observed with S1P pre-treatment in the wild-type hearts was absent in the knockout hearts (35 ± 4%, *p* = ns vs control hearts) (Fig. 2).

Of note, the present experiments were conducted concomitantly with our other experiments exploring the cardioprotective effect of S1P as a postconditioning agent. The infarct size for the control groups only [in both wild-type (*n* = 10) and knockout animals (*n* = 8)] contributed to data already reported.24

## Inhibition of STAT-3 activation abrogated protection by S1P-induced preconditioning

**Fig. 3.**
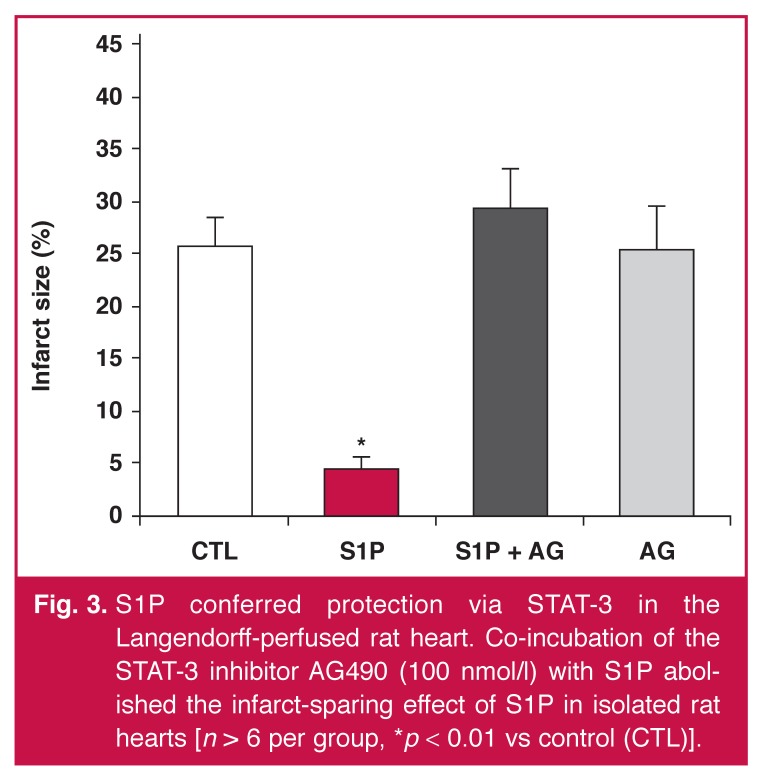
S1P conferred protection via STAT-3 in the Langendorff-perfused rat heart. Co-incubation of the STAT-3 inhibitor AG490 (100 nmol/l) with S1P abolished the infarct-sparing effect of S1P in isolated rat hearts [*n* > 6 per group, *p < 0.01 vs control (CTL)].

In the isolated rat heart model, the control hearts subjected to a regional ischaemia–reperfusion insult had an infarct size of 26 ± 8%. Pre-treatment with S1P (10 nmol/l) (Fig. 3) reduced the 40 infarct size (5 ± 3% vs ischaemic control, *p* < 0.01, *n* = 6).

To investigate the role of STAT-3 in S1P-induced preconditioning, we administered the Jak/STAT-3 inhibitor, AG490 (Fig. 3). Perfusion of AG490 abolished the cardioprotective effect of S1P (30 ± 10% vs ischaemic control, *p* = ns, *n* = 6). There was no significant difference in the size of the area at risk between the four groups (data not shown).

After 30 minutes of regional ischaemia and 120 minutes of reperfusion, the LVDP, heart rate and coronary flow were not significantly different between the four groups (Table 1). No significant differences in heart rate were found within any group at the different time points measured.

**Table 1:** Haemodynamic parameters of isolated rat hearts exposed to regional ischaemia and S1P-induced preconditioning

*Parameters*	*Preischaemia*	*Ischaemia (5 min)*	*Reperfusion (5 min)*	*Reperfusion (120 min)*
LVDP (mmHg)				
IC	86 ± 7	54 ± 10	69 ± 8	46 ± 8*
S1P	83 ± 5	35 ± 12	71 ± 7	45 ± 7*
S1P + AG	99 ± 3	65 ± 15	81 ± 3	67 ± 3*
AG	92 ± 5	57 ± 17	75 ± 4	66 ± 4*
Heart rate (bpm)				
IC	287 ± 18	263 ± 43	270 ± 14	293 ± 11
S1P	280 ± 20	250 ± 55	288 ± 42	268 ± 28
S1P + AG	273 ± 17	290 ± 60	297 ± 18	283 ± 21
AG	293 ± 18	270 ± 64	240 ± 15	257 ± 24
Coronary flow (ml/min)				
IC	10.8 ± 1.4	8 ± 5	11.2 ± 1.7	7.8 ± 1.9
S1P	9.7 ± 0.9	4 ± 1	9.8 ± 0.8	5.9 ± 0.8*
S1P + AG	9.8 ± 1.0	5 ± 1	8.4 ± 0.6	5.8 ± 0.7*
AG	8.1 ± 0.3	5 ± 1	8.2 ± 0.2	5.0 ± 0.2*

Parameters measured prior to ischaemia (pre-ischaemia), at five minutes into ischaemia and at five minutes and 120 minutes after reperfusion, respectively. IC = ischaemic control, S1P = sphingosine-1-phosphate, AG = AG490, LVDP = left ventricular developed pressure, (*n* = 6 per group.*p < 0.05 reperfusion at 120 minutes vs pre-ischaemia).

As expected, all groups showed a significant decrease (*p* < 0.05) in LVDP by the end of the reperfusion period compared to pre-ischemic values. Interestingly, only groups treated with AG490 demonstrated a significant decrease in LVDP five minutes into reperfusion compared to baseline values (*p* < 0.05). All groups except the control group demonstrated a significantly decreased coronary flow rate by the end of reperfusion compared to baseline values (*p* < 0.05).

## S1P induced an increase in phosphorylated STAT-3 in the nucleus and mitochondrion

**Fig. 4.**
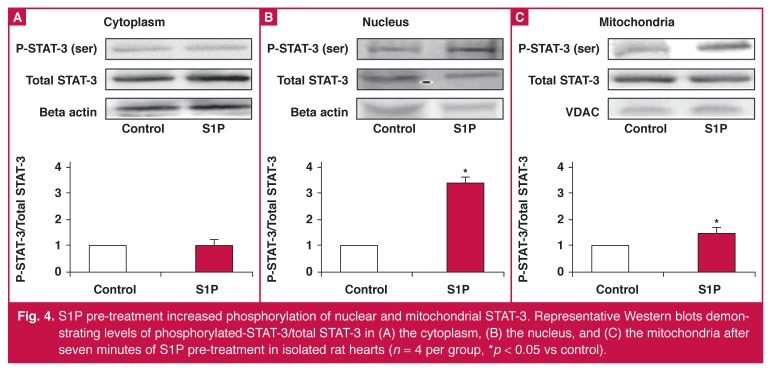
S1P pre-treatment increased phosphorylation of nuclear and mitochondrial STAT-3. Representative Western blots demonstrating levels of phosphorylated-STAT-3/total STAT-3 in (A) the cytoplasm, (B) the nucleus, and (C) the mitochondria after seven minutes of S1P pre-treatment in isolated rat hearts (*n* = 4 per group, *p < 0.05 vs control).

Western blot analysis of tissue extracted from isolated rat hearts revealed an increase in nuclear (control 1 vs S1P; 3.42 ± 0.83 arbitrary units, *p* < 0.05) and mitochondrial (control 1 vs S1P; 1.52 ± 0.10 arbitrary units, *p* < 0.05) phosphorylated/total STAT-3 after S1P pre-treatment (Fig. 4B, C). S1P pre-treatment did not significantly alter the cytoplasmic phosphorylation of STAT-3 (control 1 vs S1P; 1.00 ± 0.27 arbitrary units, *p* = ns). There was no significant change in total STAT-3 in the cytosolic, nuclear or mitochondrial fractions.

## Discussion

Our present study demonstrates that pre-treatment with S1P protected against ischaemia–reperfusion injury via the activation of STAT-3. This was evidenced by several of our findings. Firstly, S1P-induced preconditioning was inhibited in STAT-3 knockout mice. Secondly, S1P-induced preconditioning was inhibited by the STAT-3 inhibitor, AG490. Thirdly, S1P upregulated the phosphorylation of both nuclear and mitochondrial STAT-3.

## S1P can activate the JAK/ STAT-3 pathway

S1P is now recognised as a cardioprotective agent both *in vivo* and *ex vivo*.^17^,^18^,^29^,^30^ S1P can induce cardioprotection as a pre- or postconditioning stimulus.^14^,^17^,^18^,^31^ Furthermore, S1P mediates the cardioprotective effects of other preconditioning agents, e.g. TNFα,4 and ethanolamine.^9^ In fact, TNFα and STAT-3 are both members of the cardioprotective SAFE pathway and S1P may act via TNFα to activate STAT-3.^24^

Using a cardiomyocyte-specific STAT-3 knockout mouse model and the STAT-3 inhibitor, we demonstrated the requirement of STAT-3 for S1P-induced preconditioning in a whole-organ model. Although STAT-3 in other cell types of the heart has also been implicated in ischaemic preconditioning, the current results suggest that cardiomyocyte STAT-3 is required for S1P-induced cardioprotection. This is supported by experiments looking at ischaemic preconditioning, which showed that part of the protective response mediated by endothelial STAT-3 was caused by upregulation of cardiomyocyte-specific STAT-3.^32^ Less evidence is available on the preconditioning role of STAT-3 in other cardiac cell types.

## Cellular localisation of STAT-3 activation

S1P pre-treatment significantly increased nuclear levels of phosphorylated STAT-3. Phosphorylation of STAT-3 is suggested to increase translocation of STAT-3 from the cytoplasm to the nucleus where it acts as a transcription factor. However, if STAT-3 did translocate from the cytoplasm to the nucleus, one would expect a concomitant increase in total STAT-3 in the nucleus and possibly a decrease in total cytoplasmic STAT-3.

Our results do not show an increase in total nuclear STAT-3 or a decrease in cytosolic STAT-3. This may suggest either that an increase in STAT-3 export from the nucleus to the cytoplasm compensates for the movement of the phosphorylated form of STAT-3 into the nucleus, and/or that phosphorylation occurs for STAT-3 already present in the nucleus.

STAT-3 is best known as a transcription factor, however, the results of transcription are unlikely to produce the protective effects seen in these short-term experiments. This may suggest that phosphorylated STAT-3 also plays a non-transcriptional role in the nucleus, e.g. DNA repair in response to oxidative stress,^33^ or interaction with other signalling molecules within the nucleus.^34^

S1P pre-treatment also significantly increased mitochondrial levels of phosphorylated STAT-3. Recently, it has been suggested that rather than the cytosolic or nuclear pool of STAT-3 accounting for the protective effects of pre- and postconditioning, the mitochondrial pool of STAT-3 may also be important.^35^ The mechanism by which the mitochondrial STAT-3 acts remains unknown. However evidence from other studies suggests that it may affect cellular respiration and opening of the mitochondrial permeability transition pore. 23,35,36

The dual site activation of STAT-3 is in agreement with the findings of Somers *et al*.,^24^ who found that S1P-induced postconditioning caused an increase in STAT-3 activation in the nucleus and mitochondrion. Despite these similar findings, Somers *et al*.^24^ observed a concurrent decrease in cytosolic STAT-3 activation, which was not seen in the present study. The main difference in the protocols used in these two studies was the time at which S1P was administered. In S1P-induced preconditioning, S1P was administered before ischaemia to a healthy heart under physiological conditions. In S1P-induced postconditioning, the stimulus was provided in a pathological (post-ischaemic) state.

The reduction of infarct size seen in the current study was similar to that seen when S1P was given as a postconditioning agent. This may suggest that the levels of STAT-3 activation in the cytosolic fraction do not affect S1P-mediated protection but it is possible that they could affect long-term recovery from cardiovascular disease, such as remodelling.

However, it should be noted that the changes in activation of STAT-3 seen in this study focused on phosphorylation levels seven minutes after S1P treatment, which may not be representative of the changes over time. Furthermore, the current study only looked at phosphorylation of the serine residue of STAT-3. Future studies should explore the changes in phosphorylation of STAT-3 on both the serine and tyrosine residues over time in response to S1P-induced pre- and postconditioning to confirm different patterns of activation.

In humans with myocardial infarction, other cardiovascular risk factors are normally present, such as hypertension and diabetes. These may affect the ability of some pharmacological agents to protect the heart.^37^ The experiments described in this article were carried out on healthy animals. Therefore, it is imperative that S1P-induced preconditioning be confirmed in animal models that include these co-morbidities.

## Conclusion

Our data strongly suggest that the cardioprotective effects of S1P-induced preconditioning may be mediated by dual activation of STAT-3 in the nucleus and mitochondria. Our data provide a unique therapeutic opportunity to target survival against ischaemia–reperfusion injuries, especially since S1P and its sphingolipid pathway form part of the high-density lipoproteins (HDL). Addition of S1P to already existing synthetic HDL may be considered a therapeutic option in the prevention of cardiac damage associated with ischaemia–reperfusion.
